# Rapid Fabrication of Starch–Humic Acid Composite Hydrogel via an Internal Mixer for Dye Adsorption

**DOI:** 10.3390/polym18131605

**Published:** 2026-06-28

**Authors:** Xiaoyu Chen, Ao Cheng

**Affiliations:** School of Material Engineering, Jinling Institute of Technology, Nanjing 211169, China

**Keywords:** starch, polyacrylamide, internal mixer, composite hydrogel, dye adsorption

## Abstract

To develop a rapid, scalable, and eco-friendly hydrogel for wastewater decontamination, an industrial internal mixer was employed to fabricate a green hydrogel adsorbent based on starch and humic acid. Starch-grafted polyacrylamide hydrogels incorporated with humic acid were rapidly synthesized within 5 min using an internal mixer as the reactor. Starch was gelatinized in situ, followed by graft polymerization with acrylamide via free-radical polymerization and cross-linking with N,N′-methylenebisacrylamide in the same reactor. Humic acid was introduced as a natural modifier to boost the dye adsorption performance of the starch-based hydrogel. The adsorption capacity for methylene blue was evaluated under different humic acid dosages, and the maximum adsorption capacity occurred at a humic acid dosage of 20 g. Additional batch experiments revealed that the adsorption capacity increased with increasing the solution pH (from 22.6 to 54.0 mg g^−1^ over pH 4–12) and the initial dye concentration (from 14.6 to 34.1 mg g^−1^ over 40–200 mg L^−1^), while it decreased with increasing the adsorbent dosage (from 35.9 to 10.6 mg g^−1^ over 0.1–0.9 g). Distinguished from conventional laboratory stirred reactors that require 2–3 h for hydrogel synthesis, the internal mixer achieves a one-pot synthesis within 5 min, showing outstanding potential for industrial large-scale production. This work provides a time-efficient, industrially compatible strategy to prepare eco-friendly starch–humic acid hydrogels, which show promising potential as sustainable adsorbents for dye-contaminated wastewater treatment.

## 1. Introduction

Cationic dyes, such as methylene blue, are toxic, non-biodegradable, and persist in water even at low concentrations, posing significant risks to aquatic life [1,2]. Conventional removal techniques—including coagulation and adsorption on activated carbon—are often costly or low-efficiency [3]. Hydrogels have emerged as promising adsorbents due to their high specific surface area, tunable network structure, and presence of functional groups (e.g., carboxyl) that can bind cationic dyes [4,5]. As typical biopolymer adsorbents, starch-based hydrogels have attracted extensive research attention in recent years for dye wastewater treatment owing to their biodegradability, low cost, and abundant raw material sources. A large number of studies have verified their excellent removal performance towards various cationic and anionic dyes in water [6,7,8,9].

However, most starch-based hydrogels are synthesized in conventional laboratory reactors with blade stirrers, which suffer from long reaction cycles, low single-batch output, and difficulty in industrial scale-up [10,11].

This bottleneck severely restricts the practical engineering application of biopolymer hydrogel adsorbents. Hydrogels are typically prepared in laboratories, involving long production times and low yields, making them unsuitable for industrial-scale manufacturing. This challenge in large-scale production and utilization limits their practical application. Exploring how to leverage industrial equipment to produce novel adsorbent materials presents an interesting research topic. Notably, the primary limitation of the existing starch-based hydrogel adsorbents is their extremely low production efficiency rather than insufficient adsorption capacity. Most reported starch-based hydrogels deliver a similar MB adsorption capacity to one another; few studies have focused on resolving the time-consuming preparation process that blocks industrial translation.

An internal mixer is a key piece of industrial equipment primarily used for the mixing and plastication of polymer materials, such as rubber and plastics. Its production capacity spans a wide range, with a single batch output ranging from tens to hundreds of kilograms. It is suitable for large-scale continuous production, enabling the efficient manufacturing of uniformly mixed materials in bulk for industrial applications. An internal mixer typically consists of two counter-rotating rotors that turn at different speeds inside a sealed cavity, and can offer intense shear and excellent mixing capability.

Starch consists mainly of α-D-glucopyranose units linked by glycosidic bonds [12]. Besides being a food source, it also serves as a precursor for starch-based materials, which are synthesized through reactions involving its hydroxyl groups and various chemical agents [13]. Starch-based hydrogels, in particular, are prepared by graft polymerizing vinyl monomers (such as acrylic acid, acrylamide) onto starch molecules, followed by cross-linking [14]. These hydrogels have been applied as water absorbents [14], fertilizer release agents [15], dye adsorbents [16], and drug controlled release carriers [17]. Several studies have demonstrated the effectiveness of starch-based hydrogels in adsorbing dyes from textile wastewater [14,16]. For instance, starch-grafted acrylic acid–acrylamide hydrogels were synthesized via aqueous solution polymerization, and their adsorption performance for malachite green was evaluated [16]. 

Conventionally, the graft polymerization used in the synthesis of starch-based hydrogels is carried out in a reactor equipped with a standard blade stirrer [18]. In this process, starch is first gelatinized with water under stirring to form a homogeneous gel—this facilitates the efficient chemical modification of starch. Subsequently, the gelatinized starch undergoes graft polymerization with vinyl monomers to form a hydrogel [10]. Extensive time is needed for water to diffuse into starch particles and to induce gelatinization. After gelatinization, vinyl monomers are added to initiate graft polymerization onto the starch molecules. This graft polymerization step is also slow, often requiring several hours to complete [10,18].

Owing to the high shear forces that effectively break down starch granules, an internal mixer can fully gelatinize high-concentration starch—even with high viscosity—in a short period of time. This equipment also shortens the starch reaction time through efficient mixing. As a reactor, the cavity can be heated or cooled to control the temperature of the internal materials. For example, one study reported that a 60% corn starch–water mixture was fully gelatinized in an internal mixer in just 5 min at 80 °C and 50 rpm [19]. The HAAKE™ Rheomix 600 p internal mixer can fully gelatinize high-concentration starch in 10 min, reducing the total starch-based hydrogel preparation time from 2–3 h to 40 min [20].

Despite the advantages of internal mixers in rapid gelatinization and continuous production of starch materials, current research almost exclusively focuses on food processing and polymer modification. To date, there are no studies in the literature reporting the preparation of starch-based composite hydrogel adsorbents for dye removal using internal mixers. Meanwhile, humic acid, a natural organic polymer rich in active functional groups, has been proven to significantly improve the adsorption capacity of starch-based hydrogels [21,22]. Combining internal mixer technology with humic acid modification is expected to break the long-standing limitation of the low production efficiency of traditional starch-based adsorbents and to develop a novel route for the rapid industrial preparation of eco-friendly dye adsorbents.

Methylene blue (MB) is a typical toxic cationic dye widely discharged from the printing and dyeing, papermaking, and textile industries. It has strong chemical stability, poor biodegradability, and can cause serious damage to aquatic ecosystems and human health even at low concentrations [2,3].

Moreover, the detection method of methylene blue based on UV-visible spectrophotometry is mature, with stable standard curve and reliable test data. Therefore, methylene blue was selected as the model dye to evaluate the adsorption performance of the prepared hydrogel in this study.

To date, no studies have reported the preparation of starch-based adsorbents using an internal mixer. In this work, a starch–humic acid hydrogel was prepared by an internal mixer at laboratory-scale, which operates on the same working principle as its industrial counterpart. Humic acid is a natural polymer primarily composed of aromatic and alicyclic rings, with functional groups including hydroxyl, carboxyl, carbonyl, methoxy, and phenolic groups [21]. These properties make it effective in applications ranging from wastewater treatment and exhaust gas purification to soil remediation, particularly in water treatment [22]. However, humic acid exists as a powder that either disperses or partially dissolves in water, limiting its application in aqueous adsorption processes.

In this study, corn starch was first rapidly gelatinized in the internal mixer (3 min), followed by grafting with acrylamide and cross-linking with N,N′-methylenebisacrylamide in the presence of humic acid (2 min). Such a short total preparation time (5 min) has not been reported previously. The strong shear and kneading effect of the internal mixer realizes the integration of starch gelatinization, graft polymerization, and cross-linking reaction within 5 min, which is far superior to the traditional reaction process of 2–3 h. This technology is completely compatible with industrial production conditions and provides a new idea for the large-scale manufacturing of biopolymer hydrogel adsorbents. Moreover, the preparation of a starch–humic acid adsorbent material using industry equipment (internal mixer) has not been documented in the literature. This industrial equipment enables the mass production of adsorbent materials.

## 2. Materials and Methods

### 2.1. Materials and Main Instruments

N,N′-Methylenebisacrylamide (chemically pure, Shanghai Yuanju Bio-Tech Co., Ltd., Shanghai, China), acrylamide (chemically pure, Nanjing Ningshi Chemical Reagent Co., Ltd., Nanjing, China), potassium persulfate (analytical grade, Guangdong Yiming Reagent Co., Ltd., Guangzhou, China), and corn starch (chemically pure, Shandong Hengren Industry & Trade Co., Ltd., Zaozhuang, China) were used in this study. An internal mixer (model SLJ-40, Jilin University Science and Education Instrument Factory, Changchun, China) was used to prepare the composite hydrogel. The main equipment parameters of SLJ-40 are as follows: chamber volume: 270 mL; rotor speed range: 10–120 r/min; temperature control: 25–400 °C.

### 2.2. Preparation of Hydrogel

A series of hydrogels with different content of humic acid were prepared. First, 35 g of acrylamide was dissolved in 45 g of distilled water. Then, 12 g of corn starch and 10 g of humic acid were added to the aforementioned acrylamide solution and stirred to achieve uniform dispersion at room temperature. The internal mixer’s cavity was heated to 80 °C. This temperature was selected because it can ensure the efficient decomposition of the potassium persulfate initiator and complete the gelatinization of the starch granules, while avoiding high-temperature degradation of the starch and humic acid components. The liquid mixture was added to the cavity. When the temperature returned to 80 °C, the motor speed of the internal mixer was adjusted to 20 rpm, which was determined by pre-experiment to balance the mixing uniformity and shear strength, and the mixture was reacted for 3 min for starch gelatinization. A solution of 0.75 g potassium persulfate and 0.28 g N,N′-methylenebisacrylamide in 10 g distilled water was added to the cavity, and the mixture was reacted for 2 min for graft polymerization and cross-linking. During this period, the black colloid was observed to rapidly transform into a black gel. After the motor stopped, the composite hydrogel was removed from the cavity and was cut into pieces. Then the pieces were dried in an oven. To investigate the effect of humic acid content, additional hydrogels were prepared with humic acid dosages of 0 g, 15 g, 20 g, 25 g, and 30 g (while keeping other conditions constant). The hydrogels were coded as CH0 (without humic acid), CH10 (10 g humic acid), CH15 (15 g humic acid), CH20 (20 g humic acid), CH25 (25 g humic acid), and CH30 (30 g humic acid).

### 2.3. Characterization

The chemical structure of the hydrogel was characterized using a Fourier transform infrared (FTIR) spectrometer (Thermo Fisher Scientific Nicolet iS10, Waltham, MA, USA). The surface morphology of the dried hydrogels was observed using a scanning electron microscope (SEM, model Apreo 2C, Thermo Fisher Scientific, Shanghai, China). Thermo gravity (TG) was analyzed by TGA/DSC 3+ (Mettler Toledo, Greifensee, Switzerland).

### 2.4. Measurement of Swelling Degree

Dry hydrogels were cut into rice grain-sized pieces. A 1 g aliquot of these pieces was immersed in distilled water and allowed to absorb water for 36 h. The swollen hydrogel was weighed, and the swelling degree (S) was calculated using Equation (1):

(1) $$S=\;\frac{m_{2}-\;m_{1}}{m_{1}}$$

where S is the swelling degree, m_1_ is the mass of the dry hydrogel (g), and m_2_ is the mass of the swollen hydrogel (g).

### 2.5. Measurement of Adsorption Capacity

For the adsorption capacity measurements, 0.1 g of dry hydrogel was placed in a conical flask containing 50 mL of a 20 mg L^−1^ methylene blue solution. After 36 h of adsorption, the absorbance of the methylene blue solution was measured using a UV-Vis spectrophotometer (VARIAN Cary 50, Varian Australia Pty Ltd., Mulgrave, Australia). The final concentration of methylene blue (Ce) was determined from the absorbance using a pre-calibrated methylene blue standard curve, and the adsorption capacity (Q) was calculated using Equation (2):

(2) $$Q=\;\frac{\left(20-\;C_{e}\right)\;\times 0.05}{0.1}$$

where 20 is the initial concentration of methylene blue (mg L^−1^), C_e_ is the final concentration of methylene blue after adsorption (mg L^−1^), 0.05 is the volume of the methylene blue solution (L), and 0.1 is the mass of the dry hydrogel (g).

### 2.6. Adsorption Capacity at Different pH of Solution

The pH of a methylene blue solution (200 mg L^−1^) was adjusted between 4.0 and 12 by 0.1 mol L^−1^ HCl or NaOH aqueous solutions. An amount of 0.1 g CH20 was immersed into 50 mL of the above solution for 36 h at 25 °C. The adsorption capacity was calculated based on Equation (2).

### 2.7. Adsorption Capacity at Different Initial Concentration of MB

CH20 (0.1 g) was immersed into 50 mL of an MB solution at 25 °C for 36 h at initial concentrations of 200, 300, 400, 500, 600, 700, and 800 mg L^−1^, respectively. The adsorption capacity was calculated using Equation (2).

### 2.8. Adsorption Capacity at Different Dose of Adsorbent

CH20 was immersed into 50 mL of an MB solution of 200 mg L^−1^ at 25 °C for 36 h at dosages of 0.1, 0.3, 0.5, 0.7, and 0.9 g, respectively. The adsorption capacity was calculated using Equation (2).

### 2.9. Adsorption Kinetics of Composite Hydrogel in Methylene Blue Solution

An amount of 0.1 g of dry CH20 was immersed in 100 mL of a methylene blue solution of 20 mg L^−1^ at 25 °C for adsorption until adsorption equilibrium. At certain time intervals, the absorbance of residual solution was measured and the adsorption capacity was calculated.

### 2.10. Adsorption Isotherm of Composite Hydrogel in Methylene Blue Solution

An amount of 0.1 g of CH20 was immersed into 50 mL of a methylene blue solution with concentrations of 15 mg L^−1^, 20 mg L^−1^, 25 mg L^−1^, 30 mg L^−1^, and 35 mg L^−1^ at 35 °C for 36 h. The same procedure was conducted at 40 °C and 50 °C. The absorbance of solution was measured and the adsorption capacity was calculated.

### 2.11. Other Dye Adsorption

An amount of 0.1 g of CH20 was immersed in 50 mL of a methyl orange solution (concentration is 20 mg L^−1^), an acid fuchsin solution (concentration is 40 mg L^−1^), and an NaCl + methylene blue solution (concentration of methylene blue is 20 mg L^−1^, concentration of NaCl is 500 mg L^−1^), respectively. After 36 h, the absorbance of solution was measured and the adsorption capacity was calculated.

### 2.12. Reusability of the Composite Hydrogel

**Figure 1:**
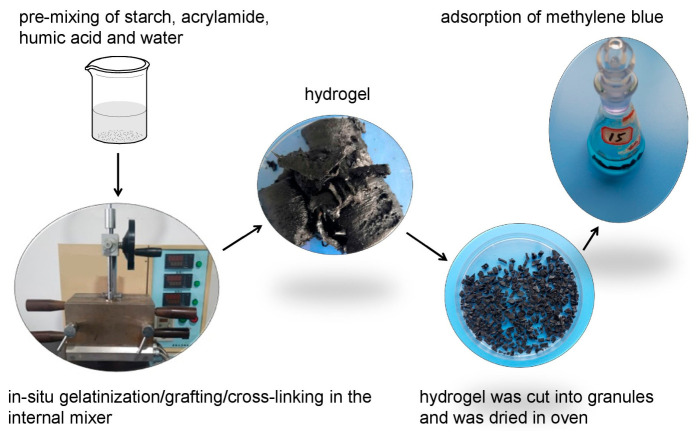
Experimental workflow for the preparation, characterization, and adsorption test of the starch–humic acid composite hydrogel.

An amount of 0.1 g of CH20 was immersed in 50 mL of 20 mg L^−1^ of a methylene blue solution at 25 °C for 36 h. After adsorption, CH20 was dipped into 0.1 mol L^−1^ of an HCl solution for 0.5 h for three times to be regenerated. Then CH20 were washed with distilled water to a neutral pH and dried in oven. CH20 were reused to adsorb methylene blue under the same adsorption conditions. Figure 1 illustrates the experimental workflow.

## 3. Results and Discussion

### 3.1. Preparation

The internal mixer was used to sequentially perform three key steps: starch gelatinization, graft polymerization, and cross-linking.

#### 3.1.1. Starch Gelatinization

In this study, starch gelatinization was completed in 3 min. Gelatinization involves the disruption of the crystalline structure within the starch granules, encompassing a sequential process of granular swelling, melting of granular crystallites, and molecular solubilization [23]. We gelatinize the starch to expose its molecular chains by breaking down the granules, enabling the efficient graft polymerization of acrylamide onto starch. These structural changes weaken the hydrogen bonds and increase the number of free hydroxyl groups, enhancing starch reactivity.

The internal mixer is equipped with counter-rotating rotors that generate high torque. The narrow gaps between the rotors and between the rotors and the cavity wall, combined with the high torque, produce intense shear stress. Shear forces break the starch granules into fragments, accelerating water penetration into the granule interior [24]. Granular crystallites are disrupted by water, and the starch molecules are solubilized. By contrast, blade stirring generates lower torque and weaker shear forces, leading to slow starch granule swelling and prolonged rupture times. Due to its limited stirring torque, blade stirring requires a large water volume to reduce the viscosity of the starch–water mixture. In conventional batch reactors, low starch concentrations (typically 3–15% in aqueous suspension) are commonly used [18]. For example, Hegazy et al. prepared a starch–g-polyacrylamide copolymer by gelatinizing 3 g of starch in 75 mL of distilled water at 100 °C for 15 min [25]. Chen et al. gelatinized 3 g of starch by dispersing it in 50 mL of water and heating the suspension to 80 °C for 1 h [26]. Zheng et al. gelatinized 1.44 g of starch in 30 mL of distilled water at 95 °C for 30 min [21].

#### 3.1.2. Graft Polymerization

**Figure 2:**
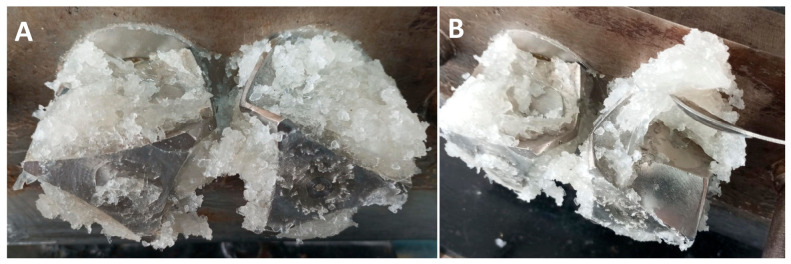
Morphology of the starch-based hydrogel prepared by graft polymerization and cross-linking reaction: (**A**) Front view; (**B**) Side view. Formula: 12 g corn starch, 35 g acrylamide, 0.75 g potassium persulfate, and 0.28 g N,N′-methylenebisacrylamide.

Following starch gelatinization, graft polymerization and cross-linking occurred sequentially and were completed rapidly within 2 min. The mixing and reaction status of the material could be observed through the internal mixer’s feed inlet. Immediately after adding the initiator and cross-linker to the cavity, the rapid formation of the starch-based hydrogel was visually confirmed. The prepared starch-based hydrogel exhibited the morphology of a mixture of strip-like shapes. This morphology results from the combined effects of the free radical polymerization-generated cross-linked network and the mechanical force field imposed by the internal mixer. The counter-rotating rotors of the mixer tear and shear the hydrogel, forming irregular fragments in the surface of the hydrogel. Some fragments appear short and strip-like due to the “tearing” action of the rotors (Figure 2).

The graft polymerization is a free radical reaction with high reaction kinetics. In this experiment, potassium persulfate (a free radical initiator) decomposes at 80 °C to generate sulfate radicals. These sulfate radicals abstract hydrogen atoms from the hydroxyl groups of starch, forming alkoxy radicals on the starch backbone [24]. Sulfate radicals primarily abstract hydrogen from the C6 hydroxyl group of starch to form alkoxy radicals. In addition to the C6 hydroxyl group, Lanthong et al. found that hydrogen atoms can be easily abstracted from weaker secondary or tertiary C-H bonds [27]. These alkoxy radicals on the starch chains then initiate the graft copolymerization of acrylamide monomers, leading to the formation of starch–g-polyacrylamide copolymers.

#### 3.1.3. Cross-Linking Reaction

Cross-linking occurred simultaneously with graft polymerization. The cross-linker, N,N′-methylenebisacrylamide, contains a polymerizable acrylamide double bond (-CH=CH-CO-NH-) at each end; these double bonds can undergo free radical polymerization with the double bonds in polyacrylamide segments or active sites on starch graft chains. When N,N′-methylenebisacrylamide interacts with polyacrylamide chains in the graft copolymer, the double bonds at its two ends undergo additional polymerization with two different grafted polyacrylamide chains attached to the starch backbone. This forms a “bridge bond” structure that connects the previously independent molecular chains. This bridging effect gradually constructs a three-dimensional network, transforming the system from a linear or branched structure to a cross-linked network. As the reaction proceeds, the N,N′-methylenebisacrylamide molecules continuously connect more molecular chains, eventually forming a three-dimensional cross-linked network throughout the entire system.

In addition to cross-linking, the internal mixer exerts a crushing effect on the hydrogel. The two counter-rotating rotors form a shear force field: when the hydrogel passes through the rotor gap, its upper and lower surfaces experience opposite frictional forces, generating an internal shear stress that causes elastic deformation of the hydrogel network. The narrow gap between the rotor and the cavity wall forms a “shear slit”. When the shear force exceeds the physical interaction strength of the hydrogel network, entanglement or weak cross-linking points between some molecular chains break, initiating network disintegration. Thus, the shearing and extrusion forces of the internal mixer can disrupt some physical interactions and weak chemical cross-links.

#### 3.1.4. Analysis the Reasons for the Fast Reaction

Potassium persulfate decomposes effectively at temperatures above 60 °C. In this experiment, the persulfate initiator decomposes at 80 °C to generate sulfate anion radicals, as described by the following reaction: K_2_S_2_O_8_ → 2K^+^ + 2SO_4_^−^. As a free radical initiator, the decomposition rate of potassium persulfate increases significantly with temperature. Higher temperatures accelerate its decomposition, increasing the initial concentration of free radicals. This, in turn, speeds up the chain initiation and propagation steps of acrylamide polymerization, enhancing the overall polymerization rate.

The initiator and cross-linker were uniformly mixed with the gelatinized starch via the kneading action of the two rotors. The rotors rotate in opposite directions, causing materials wrapped around each rotor to converge and intensively mix at their upper intersection—a process known as kneading. Their relative rotation exerts kneading and tearing forces on the material, facilitating uniform mixing. Compared to conventional blade stirrers, this efficient mixing action reduces the total preparation time from 2–3 h to just 5 min.

**Figure 3:**
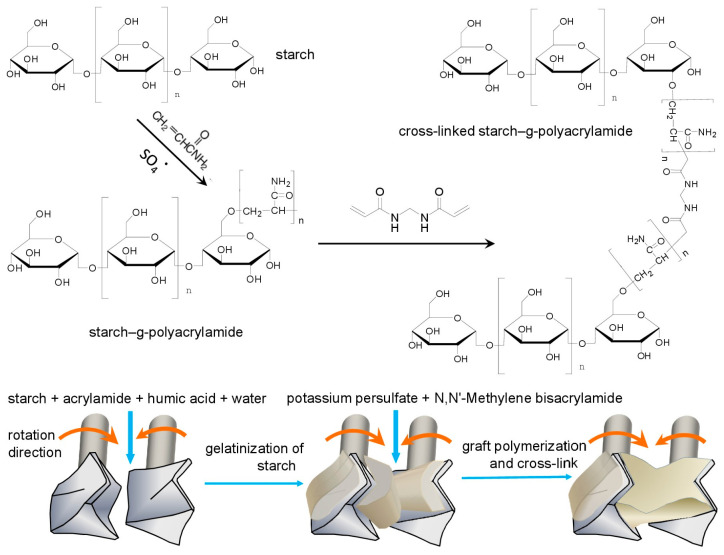
Schematic diagram of the preparation and reaction mechanism of starch–humic acid composite hydrogel.

The auto-acceleration effect (also called the gel effect) further accelerates the reaction and shortens the preparation time [28]. During polymerization, as monomers and initiators are consumed, the polymerization rate does not decrease but instead increases—a phenomenon defined as the auto-acceleration effect. Based on the micro-kinetic equation for initiator-induced free radical polymerization, the polymerization rate is proportional to the square root of the initiator concentration and the first power of the monomer concentration. Thus, high initiator and monomer concentrations lead to a high polymerization rate. The gelatinized starch has high viscosity, and the viscosity increases further as the reaction proceeds. High viscosity inhibits the termination of chain radicals, maintaining a relatively high concentration of chain radicals. However, the viscosity is not high enough to hinder the diffusion and transfer of small-molecule monomers, resulting in accelerated polymerization in the system. As the reaction continues, the viscosity of the reaction system increases until it impairs the mass transfer and diffusion of monomers, causing the polymerization rate to decrease until the reaction terminates (Figure 3).

### 3.2. FTIR Spectra and SEM Analysis

**Figure 4:**
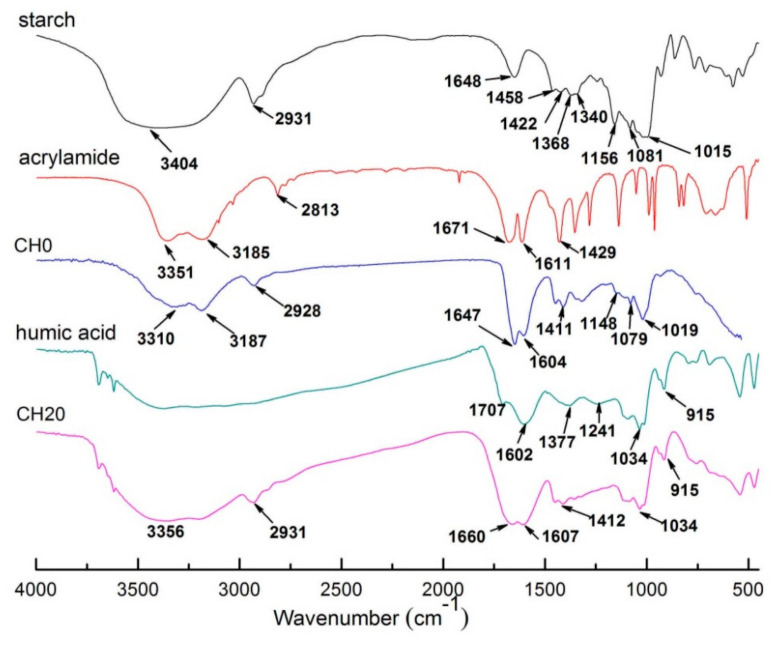
FTIR spectra of pure starch, pure acrylamide, CH0 (hydrogel without humic acid), pure humic acid, and CH20 (optimal composite hydrogel).

Figure 4 presents the FTIR spectra of starch, acrylamide, the starch-based hydrogel (without humic acid), humic acid, and CH20. Starch exhibits O-H stretching at 3404 cm^−1^, C-H stretching at 2931 cm^−1^, and C-O-C stretching at 1158, 1081, and 1015 cm^−1^ (a characteristic triplet peak of starch) [7]. Its C-H bending vibrations (e.g., -CH_2_- wagging, -CH- bending) appear at 1458, 1422, 1368, and 1340 cm^−1^, while the peak at 1648 cm^−1^ corresponds to the O-H bending vibration of bound water.

Acrylamide shows N-H stretching at 3351 and 3185 cm^−1^, C=O stretching at 1671 cm^−1^, and N-H bending of amide groups at 1611 cm^−1^—all characteristic of its CONH_2_ group. The peak at 1429 cm^−1^ corresponds to C-N stretching vibrations.

In the starch-based hydrogel spectrum, the peak at 2928 cm^−1^ arises from -CH_2_- stretching vibrations of polymerized acrylamide. Peaks at 1647 cm^−1^ correspond to C=O stretching in -CONH_2_, and peaks at 1604 cm^−1^ correspond to N-H bending vibrations [29]. These results confirm the successful graft polymerization of acrylamide onto starch.

In the humic acid spectrum, the absorption peak at 1707 cm^−1^ corresponds to the C=O stretching vibration of the carboxylic group [30]. Other absorption peaks include 1602 cm^−1^ (asymmetric stretching of –COO^−^ in humic acid), 1241 cm^−1^ (phenolic C–O stretching in humic acid) [31], 1034 cm^−1^ (antisymmetric stretching vibration of Si-O), and 915 cm^−1^ (out-of-plane bending vibration of C-H bonds on aromatic rings).

In the CH20 spectrum, peaks characteristic of both the starch-based hydrogel (2931, 1660, 1607, 1412 cm^−1^) and humic acid (1034, 915 cm^−1^) are observed, which suggests the successful incorporation of humic acid into the starch-based hydrogel matrix as a proposed composite structure.

**Figure 5:**
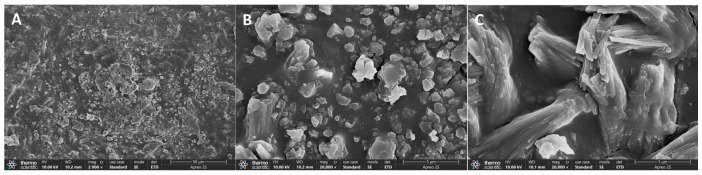
SEM images of dried CH20 composite hydrogel. (**A**) Magnification: 2000×, scale bar = 50 μm; (**B**) Magnification: 20,000×, scale bar = 5 μm; (**C**) Magnification: 20,000×, scale bar = 5 μm.

Figure 5A shows the macroscopic morphology of the dried composite hydrogel (2000× magnification). The composite gel surface presents an obvious rough structure, with a large number of irregular micron-sized particles uniformly distributed. It is hypothesized that humic acid molecules form aggregated particles through hydrogen bonds and van der Waals forces, and the rough surface structure can effectively increase the contact area between the hydrogel and dye solution, potentially providing abundant exposed adsorption active sites for methylene blue molecules. Figure 5B (20,000× magnification) shows that most humic acid aggregates are 1–5 μm in size, which are closely combined with the starch-based hydrogel matrix without any obvious gaps, implying favorable interfacial compatibility between humic acid and the starch–polyacrylamide substrate. Figure 5C displays the fibrous stacked structure of the silicate components in humic acid. The inorganic silicate forms rigid particles, which are wrapped by the shrinking organic phase during drying to construct a “rigid-flexible” composite structure, which may help improve the mechanical strength of the dried hydrogel. The rough surface observed by SEM and the three-dimensional network structure can provide effective diffusion channels and a partial specific surface area for dye molecules.

The BET specific surface area of CH20 is 17.8 m^2^/g, which belongs to the typical range of biopolymer hydrogels without a special pore-forming treatment. The nitrogen adsorption–desorption isotherm of the CH20 starch–humic acid composite hydrogel belonged to the typical IUPAC type IV isotherm with an H3-type hysteresis loop. Nearly no nitrogen adsorption was observed at low relative pressure (*p*/*p*_0_ < 0.1), verifying the extremely low proportion of micropores in the material. The gradual adsorption rise in the medium relative pressure region reflected multilayer gas adsorption in mesoporous channels, while the sharp adsorption increase at (*p*/*p*_0_ > 0.9) was attributed to macropores and external surface adsorption induced by the aggregation of humic acid microparticles. The BET specific surface area of CH20 was 17.8 m^2^ g^−1^, accompanied by a total pore volume of 0.078 cm^3^ g^−1^ and a BJH average pore diameter of 18.6 nm. The pore size distribution results indicated that mesopores (2–50 nm) were dominant, with a pore volume contribution of 82.7%, and the most probable pore size was 16.2 nm, whereas micropores only occupied 2.1% of the total pore volume.

### 3.3. Thermogravimetric Analysis (TGA)

**Figure 6:**
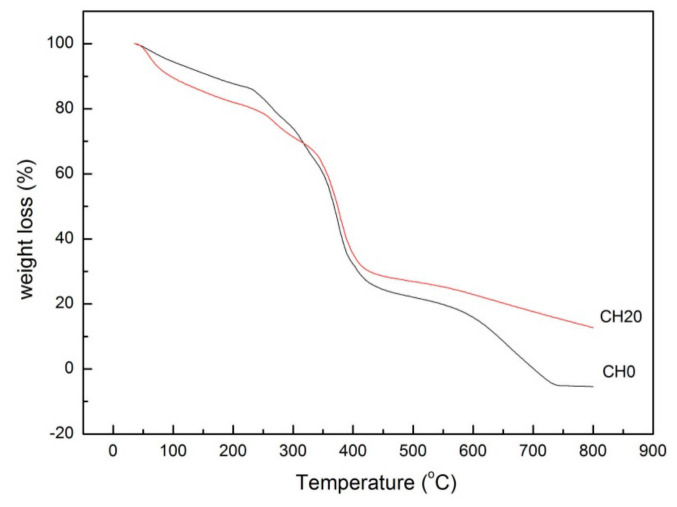
TGA curves of CH0 (hydrogel without humic acid) and CH20 (optimal composite hydrogel).

Figure 6 shows the thermogravimetric analysis (TGA) curves for the CH20 and CH0 (without humic acid).

CH0: The overall mass loss persists in the high-temperature region (e.g., above 700 °C), with relatively little residual mass remaining at the end. This indicates that the cross-linked starch-grafted polyacrylamide product has relatively limited thermal stability and is more susceptible to decomposition and other mass-loss processes at elevated temperatures.

CH20: The curve differs from the starch-based hydrogel in aspects like mass loss rate during the low-temperature stage (early phase). At higher temperatures, it shows less mass loss and more final residual mass. This demonstrates that adding humic acid enhances the thermal stability of the cross-linked starch-grafted polyacrylamide, enabling it to retain more mass at higher temperatures. The CH20 hydrogel thus exhibits better high-temperature resistance and is less prone to thermal decomposition.

The improved thermal stability of the composite hydrogel is presumed to originate from strong intermolecular interactions between humic acid and the starch–polyacrylamide network. A large number of hydroxyl, carboxyl, and phenolic hydroxyl groups on humic acid may form hydrogen bonds with the functional groups of the hydrogel matrix. These hypothesized interactions restrict the movement of polymer molecular chains and potentially inhibit the thermal decomposition reaction of the network at a high temperature. Therefore, the addition of humic acid is proposed to effectively optimize the thermal resistance of the starch-based hydrogel.

### 3.4. Swelling Degrees of Hydrogels

**Figure 7:**
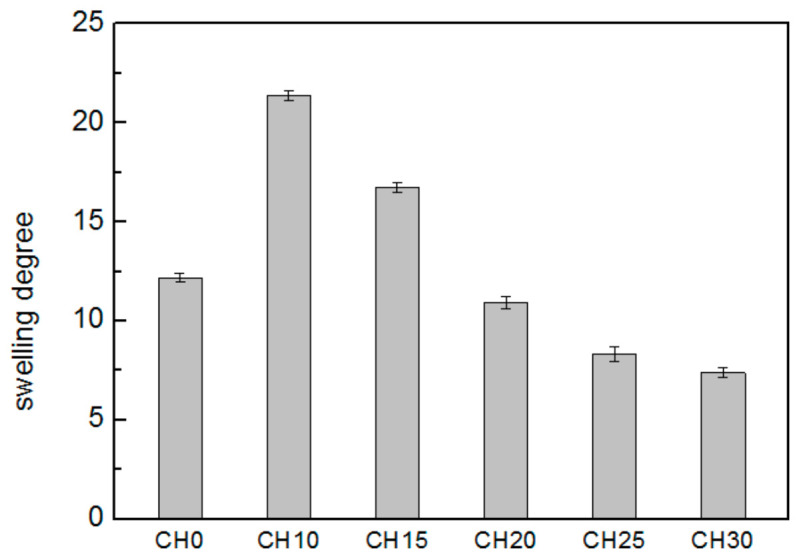
Swelling degrees of composite hydrogels with different humic acid dosages (CH0–CH30).

A higher swelling degree promotes the diffusion of dye solutions into the hydrogel. The swelling degrees of hydrogels with different humic acid contents are shown in Figure 7. CH10 (containing 10 g of humic acid) exhibits the highest swelling degree (19.27). This indicates that a lower humic acid content promotes water absorption, while higher contents reduce the water absorption. Humic acid contains hydrophilic functional groups (e.g., carboxyl, hydroxyl groups) that can bind water molecules—thus, increasing the humic acid content initially enhances water absorption. However, excess humic acid acts as filler, occupying the network space available for water retention and reducing the swelling degree.

### 3.5. Adsorption Capacity of Hydrogels

**Figure 8:**
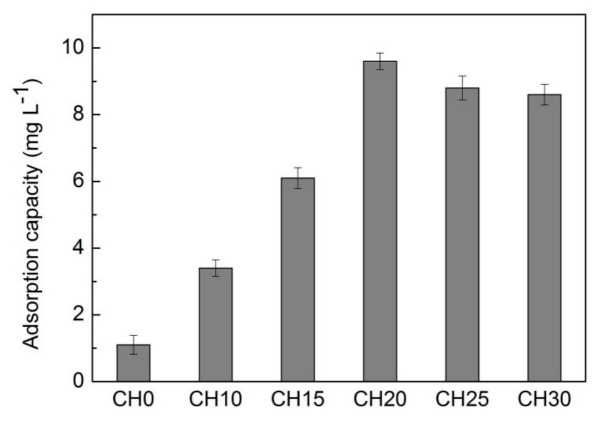
Adsorption capacities of composite hydrogels with different humic acid dosages for methylene blue.

For hydrogels, increasing the humic acid content initially enhances methylene blue adsorption; however, excess humic acid inhibits the diffusion of the methylene blue solution into the hydrogel, reducing the adsorption capacity (Figure 8). High adsorption capacities were observed when the humic acid dosage exceeded 15 g (specifically at 20 g, 25 g, and 30 g). Among these, CH20 (20 g humic acid) exhibited the largest adsorption capacity (9.6 mg g^−1^). Humic acid contains active functional groups (e.g., carboxyl, hydroxyl, and phenolic hydroxyl groups). The anionic carboxylate groups (-COO^−^) of humic acid can form ionic complexes with the cationic imino groups (-NH_2_^+^) of methylene blue, enabling efficient adsorption. As a natural product, humic acid offers unique advantages for dye adsorption from wastewater: it is environmentally friendly, readily available, and biocompatible, with inherent functional groups enabling efficient adsorption without causing secondary pollution.

From Figure 7 and Figure 8, we can see that the optimal humic acid content for swelling (10 g, with a swelling degree of 19.27) does not align with the optimal content for methylene blue adsorption (exceeding 15 g, specifically 20 g, 25 g, and 30 g, with CH20 showing the most pronounced effect).

For hydrogel with low content humic acid, such as CH10, more network space is available for water absorption (swelling degree = 21.4), but the limited number of adsorption sites restricts methylene blue removal. By contrast, for composite hydrogels with high content humic acid, such as CH20, CH25, CH30, the humic acid introduces additional carboxyl/hydroxyl groups (adsorption sites), which outweighs the effect of reduced swelling. For CH20, the adsorption composite reaches 9.6 mg g^−1^, even with a lower swelling degree (10.9). However, excessive humic acid inhibits the diffusion of the methylene blue solution into the hydrogel interior and reduces the adsorption capacity.

Table 1 summarizes the methylene blue adsorption performance of biopolymer hydrogels reported in recent years. As shown, the maximum adsorption capacity of our CH20 hydrogel (9.6 mg g^−1^) is comparable to conventional biopolymer adsorbents synthesized via long-duration stirred-tank reactions. It is critical to clarify that the core competitive advantage of the material developed in this work does not rely on superior adsorption capacity, but lies in its 5 min one-pot synthesis and intrinsic compatibility with industrial batch production, which delivers unique comprehensive practical merits for wastewater remediation [32,33,34].

### 3.6. Effect of pH on Adsorption Capacity

**Figure 9:**
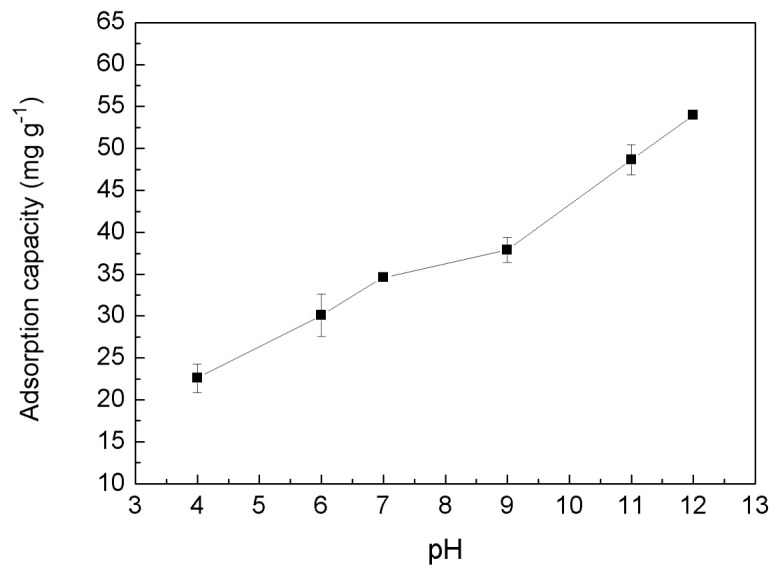
Effect of solution pH (4–12) on the methylene blue adsorption capacity of the CH20 hydrogel.

The influence of the initial solution pH on the adsorption capacity of methylene blue (MB) by the starch–humic acid hydrogel was investigated over a pH range of 4–12, as shown in the data (Figure 9). The adsorption capacity increased steadily with a rising pH, from 22.57 mg g^−1^ at pH 4 to 53.98 mg g^−1^ at pH 12. This significant enhancement under alkaline conditions is proposed to stem from stronger electrostatic interaction between the adsorbent and the cationic dye MB.

At a low pH (4–6), the abundant H^+^ ions compete with MB^+^ cations for the available binding sites on the hydrogel. Moreover, the protonation of carboxyl and phenolic hydroxyl groups originating from humic acid and hydrolyzed acrylamide units reduces the negative charge density on the adsorbent surface, thereby weakening the electrostatic attraction and resulting in lower uptake.

As the pH increased to 7 and above, deprotonation of these functional groups occurred, rendering the hydrogel surface more negatively charged. The strong electrostatic attraction between the negatively charged sites and the cationic MB molecules led to a sharp rise in adsorption capacity, reaching a maximum of 53.98 mg g^−1^ at pH 12. No significant plateau was observed within the tested range, suggesting that even a higher pH might further promote adsorption, although practical constraints (e.g., dye stability) should be considered.

In summary, the adsorption of MB onto the starch-based hydrogel is strongly pH-dependent, with alkaline conditions being highly favorable due to enhanced electrostatic interactions. This finding indicates that the prepared composite hydrogel is particularly effective for removing cationic dyes from basic wastewater.

### 3.7. Effect of Initial Methylene Blue Concentration on Adsorption Capacity

**Figure 10:**
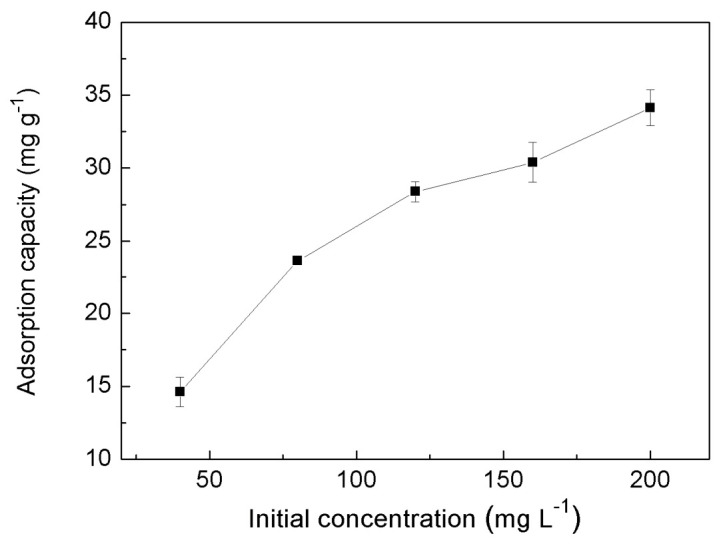
Effect of initial methylene blue concentration (40–200 mg L^−1^) on adsorption capacity of the CH20 hydrogel.

The influence of initial methylene blue (MB) concentration on the adsorption capacity of the starch-grafted polyacrylamide/humic acid hydrogel was evaluated over a range of 40–200 mg/L, with a fixed adsorbent dosage of 0.1 g. As shown in the data (Figure 10), the adsorption capacity increased progressively from 14.62 mg g^−1^ at 40 mg L^−1^ to 34.14 mg g^−1^ at 200 mg L^−1^. This positive correlation can be explained by the enhanced concentration gradient driving force at higher initial MB concentrations, which facilitates the mass transfer of dye molecules from the bulk solution to the adsorbent surface.

However, the rate of increase in the adsorption capacity diminished with a rising concentration. Specifically, raising the MB concentration from 40 to 80 mg/L (a 40 mg/L increment) resulted in an increase of approximately 9.01 mg/g, whereas the same increment from 160 to 200 mg/L yielded only a 3.75 mg/g increase. This trend suggests that the adsorbent surface was approaching saturation, as the number of available binding sites on the hydrogel became progressively limited. The observed behavior is characteristic of Langmuir-type adsorption, where the initial uptake is governed by abundant vacant sites and higher concentrations, while the capacity gradually plateaus as the active sites become occupied.

In summary, increasing the initial MB concentration enhances the adsorption capacity due to a stronger driving force, but the finite availability of binding sites eventually limits further uptake. The starch-based hydrogel exhibited effective removal at higher dye concentrations, indicating its potential for treating moderately concentrated cationic dye wastewater.

### 3.8. Effect of Adsorbent Dosage on Adsorption Capacity

**Figure 11:**
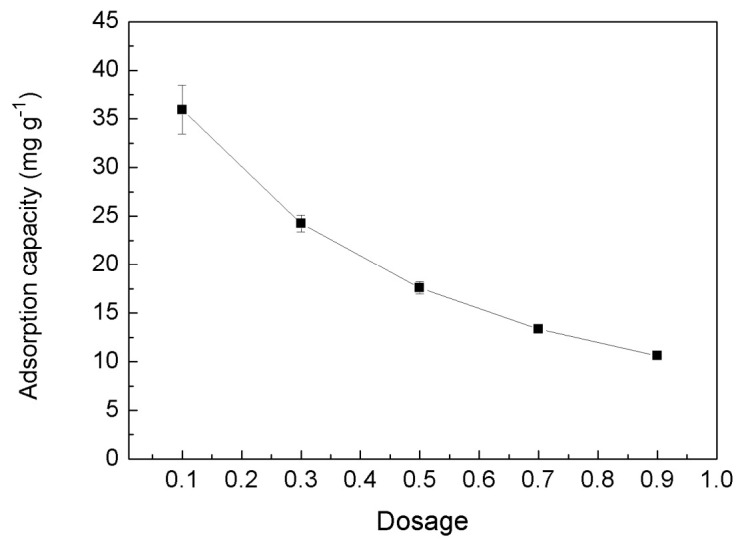
Effect of adsorbent dosage (0.1–0.9 g) on methylene blue adsorption capacity of the CH20 hydrogel.

The influence of hydrogel dosage on the adsorption capacity toward methylene blue (MB) was examined by varying the sample mass from 0.1 g to 0.9 g while keeping the initial MB concentration constant at 200 mg/L. As presented in the data (Figure 11), the adsorption capacity exhibited a pronounced decrease with the increasing adsorbent dosage, dropping from 35.95 mg/g at 0.1 g to 10.60 mg/g at 0.9 g. An adsorbent dosage of 0.1 g was optimal for maximizing the adsorption capacity of the starch-based hydrogel for MB.

This inverse relationship can be attributed to the phenomenon of active site unsaturation. At a low adsorbent dosage (0.1 g), the fixed amount of MB molecules in the solution (200 mg/L) encounters a relatively limited number of binding sites, leading to a higher dye uptake per unit mass of hydrogel. Conversely, when the dosage is increased to 0.9 g, a large excess of available sites is present relative to the total dye concentration. Consequently, many of these sites remain unoccupied, and the competition for MB molecules among the abundant particles reduces the adsorption capacity expressed on a per-gram basis.

Additionally, at higher solid loadings, particle aggregation becomes more likely, which may reduce the effective surface area and hinder mass transfer, further contributing to the observed decline in capacity. Notably, the total dye removal efficiency likely increased with the dosage, even though the capacity decreased, which is typical in batch adsorption studies.

### 3.9. Adsorption Kinetic

**Figure 12:**
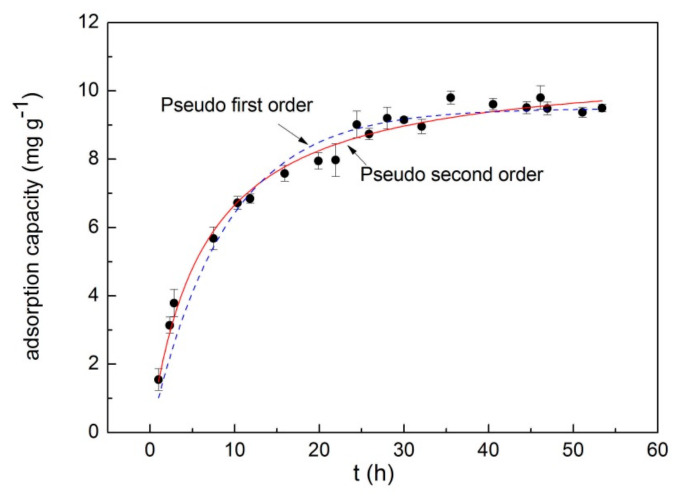
Nonlinear fitting curves of pseudo-first-order and pseudo-second-order kinetic models for methylene blue adsorption on the CH20 hydrogel.

Figure 12 illustrates the effect of contact time on the adsorption capacity of the hydrogel (CH20) at 25 °C. The adsorption capacity increased rapidly within the first 12 h, followed by a slower increase, and approached equilibrium after approximately 28 h. The experimental data were fitted using the pseudo-first-order (Equation (3)) and pseudo-second-order (Equation (4)) kinetic models. The intraparticle diffusion model and the liquid film diffusion model were supplemented to analyze the diffusion steps during adsorption.

log(*Q_eq_* − *Q_t_*) = log*Q_eq_* − *k*_1_*t* (3)

(4) $$\frac{t}{Q_{t}}=\frac{1}{k_{2}Q_{eq}^{2}}=\frac{1}{Q_{eq}}t$$

In these models, k_1_ (min^−1^) and k_2_ (g mg^−1^ min^−1^) represent the rate constants of the pseudo-first-order and pseudo-second-order models, respectively, and Q_eq_ (mg g^−1^) and Q_t_ (mg g^−1^) denote the adsorption capacities at equilibrium and at time t (min), respectively.

As shown in Figure 12, nonlinear fitting was performed for both kinetic models. Based on the higher correlation coefficient (R^2^) and lower chi-squared (χ^2^) values summarized in Table 2, the pseudo-second-order model exhibits better agreement with the experimental data. This suggests that the adsorption process is predominantly governed by chemical sorption.

**Figure 13:**
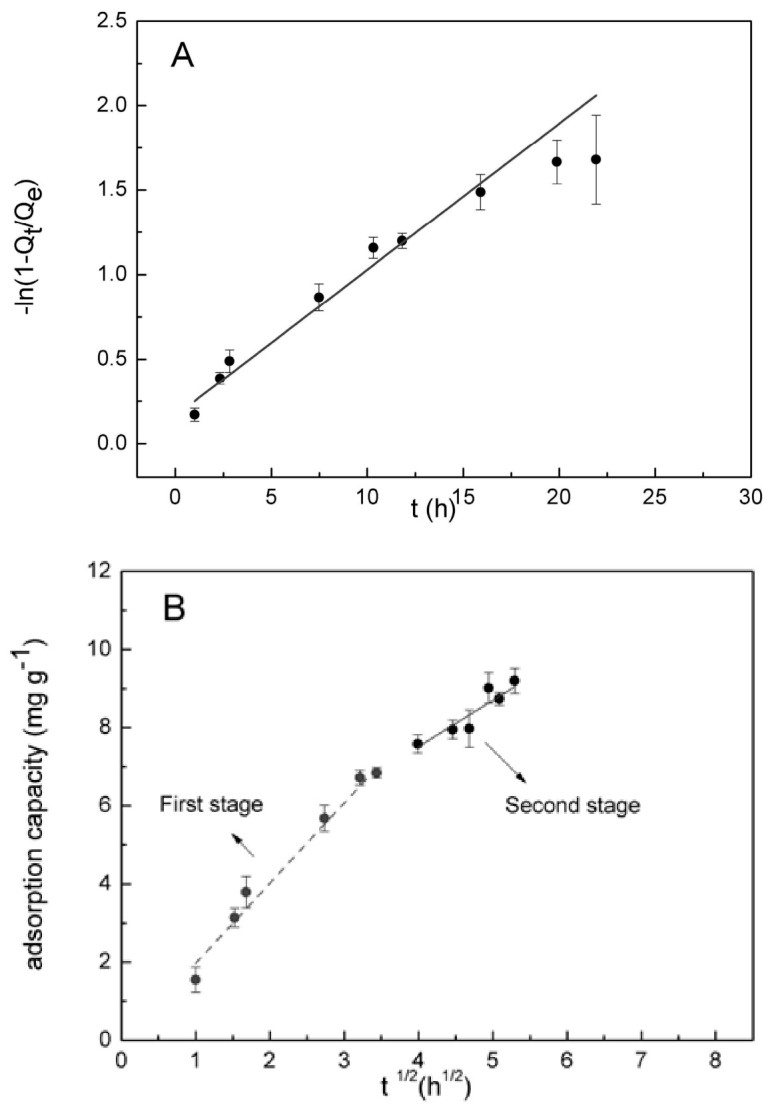
Linear fitting of liquid film diffusion (**A**) and intraparticle diffusion (**B**) for methylene blue adsorption on the CH20 hydrogel.

To further clarify the mass transfer mechanism during adsorption, two additional kinetic models, the liquid film diffusion model and the intraparticle diffusion model, were supplemented for data analysis. The linear equation of the liquid film diffusion model is expressed as follows:

(5) $$-\ln(1-\frac{Q_{t}}{Q_{e}})=k_{fd}t$$

where k_fd_ (h^−1^) is the liquid film diffusion rate constant. This model is only applicable to the initial stage of adsorption, where dye molecules diffuse through the liquid film on the hydrogel surface. The intraparticle diffusion model (Weber–Morris model) is given by the following equation:

(6) $$Q_{t}=k_{fd}t^{0.5}+C$$

where k*_id_* (mg g^−1^ h^0.5^) is the intraparticle diffusion rate constant and C stands for the intercept related to boundary layer thickness. For hydrogel adsorption systems, this model usually presents two distinct linear segments. All kinetic fitting parameters, including the correlation coefficient (R^2^), are summarized in Table 2. According to the higher (R^2^) value, the pseudo-second-order model fits the experimental data better, indicating that the adsorption process is mainly controlled by chemical adsorption. The fitting results of the liquid film diffusion and intraparticle diffusion models are presented in Figure 13. The liquid film diffusion model was fitted using data from the initial adsorption stage, and a good linear relationship was obtained. The intraparticle diffusion curve is divided into two linear regions, indicating two successive mass transfer steps. Combined with all fitting results, the whole adsorption process can be divided into three stages: the liquid film diffusion stage, the intraparticle diffusion stage, and the equilibrium stage. Liquid film diffusion stage (0–11.82 h): Methylene blue molecules rapidly pass through the surface liquid film under the concentration gradient, which is the fastest step of the whole process. Intraparticle diffusion stage (11.82–28.02 h): After crossing the surface liquid film, dye molecules gradually diffuse into the three-dimensional network structure of the hydrogel, which acts as the main rate-controlling step. Equilibrium stage (>28.02 h): The adsorption and desorption rates reach dynamic balance, and the adsorption capacity tends to be stable. The two linear segments of intraparticle diffusion and non-zero intercept prove that the adsorption process is co-controlled by liquid film diffusion and intraparticle diffusion, rather than a single diffusion step.

In addition to the pseudo-first-order and pseudo-second-order models, the intraparticle diffusion and liquid film diffusion models were supplemented to analyze the whole adsorption process [35]. Combined with the intraparticle diffusion and liquid film diffusion models, the whole adsorption process can be divided into the following three stages: (1) Liquid film diffusion stage: methylene blue molecules diffuse through the liquid film on the hydrogel surface, which is the fast adsorption stage within the first 12 h; (2) Intraparticle diffusion stage: dye molecules further diffuse into the internal network of the hydrogel, with a slower rate; (3) Equilibrium stage: the adsorption and desorption rates reach balance. The intercept of the intraparticle diffusion model does not pass through the origin, indicating that intraparticle diffusion is not the only rate-controlling step. The high fitting degree of the pseudo-second-order model proves that chemical interaction is the main controlling factor of adsorption.

From a practical engineering perspective, the 36 h adsorption equilibrium time observed in batch tests represents a notable limitation for continuous industrial wastewater treatment. The intraparticle diffusion model fitting confirms that slow intraparticle diffusion through the dense cross-linked hydrogel network acts as the primary rate-limiting factor, requiring extended contact time for MB to reach internal adsorption sites. For future industrial deployment, targeted material optimization strategies are required to shorten the equilibrium duration: reducing cross-linker loading to form looser hydrogel networks, granulating bulk hydrogel into small particles to shorten diffusion pathways, or integrating the adsorbent within fluidized-bed reactor systems to accelerate mass transfer efficiency.

### 3.10. Adsorption Isotherm

The adsorption isotherm of the hydrogel was evaluated using the Langmuir, Freundlich, D-R, and Sips models [36,37]. The thermodynamic parameters (ΔG, ΔH, ΔS) were calculated based on isotherm data to analyze the thermodynamic characteristics of adsorption. The Langmuir model, which describes monolayer adsorption on a homogeneous surface, is given by Equation (7). By contrast, the Freundlich model, represented by Equation (8), characterizes multilayer adsorption on a heterogeneous surface.

(7) $$\frac{C_{e}}{Q_{eq}}=\frac{1}{Q_{max}}C_{e}+\frac{1}{Q_{max}b}$$

(8) $$InQ_{eq}=\frac{1}{n}InC_{e}+In\;K_{F}$$

In these equations, Q_max_ (mg g^−1^) refers to the maximum adsorption capacity, b (L mg^−1^) is the Langmuir equilibrium constant, K_F_ is the Freundlich capacity parameter, and 1/n (dimensionless) represents the adsorption intensity.

**Figure 14:**
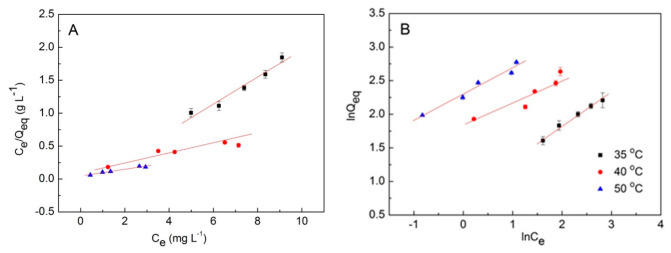
The Langmuir model (**A**) and Freundlich model (**B**) for methylene blue adsorption on the CH20 hydrogel.

The fitting curves of the Langmuir and Freundlich models are displayed in Figure 14A,B, respectively. The analysis reveals that the Freundlich model provides a better fit to the experimental data. This conclusion is supported by the higher R^2^ value listed in Table 3, confirming the suitability of the Freundlich model for describing the adsorption behavior of the hydrogel. The applicability of the Freundlich model indicates that adsorption takes place on a heterogeneous surface. This further implies that methylene blue is initially adsorbed by humic acid on the outer surface and subsequently diffuses into the interior of the hydrogel for further adsorption.

The Langmuir model was firstly fitted using the experimentally measured equilibrium concentration (Ce). The obtained maximum adsorption capacity Q_m_ was adopted as the initial value for iterative fitting of the Sips model, which is a conventional method for a nonlinear isotherm analysis in adsorption research.

The Dubinin–Radushkevich (D-R) model can be expressed as follows:

(9) $$\ln Q_{e}=\ln Q_{m}-\beta \varepsilon^{2},\;\varepsilon =RT\ln(1+\frac{1}{C_{e}})$$

The average adsorption energy is calculated by $E=\frac{1}{\sqrt{-2\beta }}$.

The Sips model can be expressed as follows:

(10) $$\ln(\frac{Q_{m}}{Q_{e}}-1)=\frac{1}{n_{s}}\ln C_{e}-\ln K_{s}$$

In these equations, Q_m_ is the maximum adsorption capacity, Ks is the equilibrium constants, and 1/n and n_s_ are the adsorption intensity and heterogeneity factor, respectively. All isotherm parameters and correlation coefficients (R^2^) are summarized in Table 3. According to the (R^2^) values, the Freundlich and Sips models show a higher fitting accuracy than the Langmuir and D-R models, indicating that the adsorption of methylene blue on the composite hydrogel occurs on a heterogeneous surface. The value of (1/n) ranges from 0.36 to 0.42 (0 < 1/n < 1), proving that the adsorption process is favorable. From the D-R model results, the average adsorption energy E is far less than 8 kJ mol^−1^ at all temperatures, demonstrating that the adsorption is dominated by physical adsorption. Combined with the thermodynamic analysis, (ΔG < 0), (ΔH > 0), and (ΔS > 0), which indicates the adsorption of methylene blue is a spontaneous and endothermic process, and higher temperature is beneficial to the adsorption reaction.

### 3.11. Other Dye Adsorption

**Figure 15:**
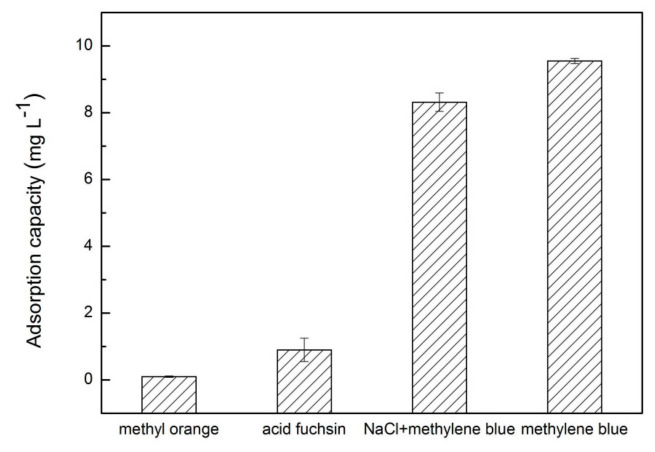
Adsorption capacities of the CH20 hydrogel for different dyes and mixed solutions (methyl orange, acid fuchsin, NaCl + methylene blue, pure methylene blue).

To avoid limiting the adsorption research to a single dye pollutant and to explore the selective adsorption performance of the CH20 hydrogel, two typical anionic dyes (methyl orange and acid fuchsin) were selected for comparative adsorption experiments with cationic methylene blue. As shown in Figure 15, the adsorption capacities of methyl orange (0.1 mg g^−1^) and acid fuchsin (0.9 mg g^−1^) are lower than methylene blue (9.6 mg g^−1^). Methyl orange and acid fuchsin are anionic dyes, whereas methylene blue is cationic. It is proposed that strong electrostatic attraction between the positively charged methylene blue molecules and the negatively charged humic acid moieties embedded in the hydrogel accounts for this distinct adsorption selectivity. The existence of NaCl (500 mg L^−1^) decreased the adsorption capacity (8.3 mg g^−1^) of the hydrogel for methylene blue. The Na^+^ ions released from NaCl compete with the cationic methylene blue for the negative adsorption sites (primarily from humic acid’s carboxyl and phenolic groups) in the hydrogel, leading to the observed decrease in adsorption capacity. This salt interference experiment further evaluated the anti-interference ability of the hydrogel in complex aqueous environments, providing preliminary support for its potential application in real dye wastewater treatment.

### 3.12. Reusability of the Hydrogel

**Figure 16:**
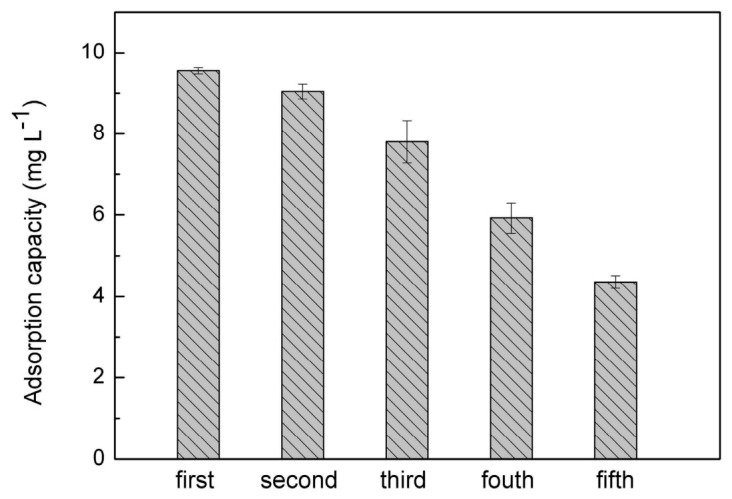
Reusability of the CH20 hydrogel after five consecutive adsorption–desorption cycles.

The reusability results are shown in Figure 16. The adsorption capacity in the second cycle (9.04 mg g^−1^) was similar to that of the first cycle (9.55 mg g^−1^), indicating that the hydrogels can be effectively regenerated while largely retaining their adsorption ability. The adsorption capacity decreased more noticeably in the fifth cycle (4.36 mg g^−1^). Overall, CH20 retained 45.6% of its initial adsorption capacity after five consecutive adsorption–desorption cycles.

### 3.13. Adsorption Mechanism

**Figure 17:**
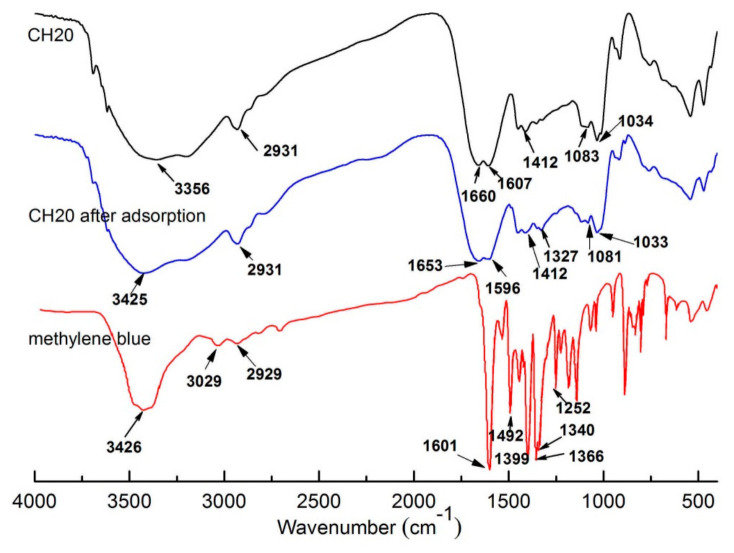
FTIR spectra of the original CH20 hydrogel, CH20 after methylene blue adsorption, and pure methylene blue.

All interaction pathways summarized below represent the proposed adsorption mechanisms deduced from the spectral and batch adsorption data. The primary mechanism for methylene blue adsorption onto the hydrogel is proposed to be the electrostatic interaction between the negatively charged functional groups of humic acid and the positively charged dye molecules. The FTIR (Figure 17) after adsorption shows a shift of the humic acid carboxylate peak (1607 cm^−1^ shifted to 1596 cm^−1^), which suggests potential binding between -COO^−^ and methylene blue’s –NH^2+^. This is further supported by the markedly different adsorption capacities observed for cationic methylene blue compared to the anionic dyes, methyl orange and acid fuchsin. The adsorption kinetics also supports this mechanism, as the process was best described by the pseudo-second-order model, which is often associated with chemisorption.

Combined with all characterization and experimental results, we hypothesize the following auxiliary adsorption pathways: (1) Dominant effect: Electrostatic interaction between anionic carboxyl/phenolic hydroxyl groups of humic acid and cationic methylene blue; (2) Auxiliary effect: Presumed hydrogen bonding between hydroxyl groups on the hydrogel network and dye molecules; (3) Physical adsorption: Rough surface and network pores provide partial physical adsorption sites. It is worth emphasizing that the core innovation of this work is the rapid preparation technology based on the industrial internal mixer, and the above mechanism analysis is only used to verify the application potential of the material.

### 3.14. Limitations of the Present Study

This investigation was carried out at laboratory scale using a SLJ-40 internal mixer, with methylene blue selected as a single model cationic dye to assess the adsorption performance. Actual industrial printing and textile wastewater contains complex mixed pollutants, including multiple coexisting dyes, inorganic salts, surfactants, and organic auxiliaries, which may exert competitive or inhibitory effects on the adsorption capacity of the starch–humic acid hydrogel. Therefore, the adsorption results obtained under simplified lab conditions cannot be directly extrapolated to full-scale industrial wastewater treatment without further validation. In addition, the as-prepared hydrogel requires roughly 36 h to reach adsorption equilibrium, which limits its application in high-throughput continuous wastewater treatment systems. Future research will focus on pilot-scale synthesis trials, real wastewater adsorption tests, and structural optimization of the hydrogel network to shorten the intraparticle diffusion time and to improve the practical engineering applicability.

## 4. Conclusions

This work successfully realized the rapid one-pot synthesis of a starch–humic acid composite hydrogel within 5 min using an industrial internal mixer, which constitutes the primary novelty of this study and completely overcomes the long 2–3 h preparation cycle limitation of traditional starch-based hydrogels manufactured via laboratory stirred reactors. The internal mixer relies on a strong shear and kneading effect to complete starch gelatinization, graft polymerization, and cross-linking reaction efficiently, and this technology is compatible with industrial large-scale batch production. A starch–humic acid hydrogel was rapidly prepared in an internal mixer within 5 min. The use of the internal mixer significantly shortened the time required for starch gelatinization, graft polymerization, and cross-linking, and enabled the mass production of the hydrogel. An optimal humic acid dosage was also identified to maximize its methylene blue adsorption capacity: high adsorption capacities were observed when the humic acid dosage exceeded 15 g (20 g, 25 g, and 30 g). Furthermore, the adsorption capacity was strongly influenced by the solution pH, initial dye concentration, and adsorbent dosage. Specifically, the capacity increased with rising pH (from 22.6 mg g^−1^ at pH 4 to 54.0 mg g^−1^ at pH 12) and with higher initial methylene blue concentrations (from 14.6 mg g^−1^ at 40 mg L^−1^ to 34.1 mg g^−1^ at 200 mg L^−1^), whereas it decreased markedly as the adsorbent dosage increased (from 35.9 mg g^−1^ at 0.1 g to 10.6 mg g^−1^ at 0.9 g). The optimal adsorbent dosage is 0.1 g for maximum unit adsorption capacity. The adsorption equilibrium and kinetics study of the composite hydrogel indicate that the adsorption behavior follows the Freundlich model and the pseudo-second-order equation. The adsorption process includes liquid film diffusion and intraparticle diffusion steps, and is a spontaneous endothermic reaction. The dye-loaded composite hydrogel could be regenerated, retaining 81.7% of its initial adsorption capacity after three cycles. As a natural product, humic acid enhances dye adsorption while ensuring environmental compatibility. The internal mixer adopted in this study is standard industrial polymer processing equipment with large single-batch output and continuous production capacity. The raw materials (corn starch, humic acid) are cheap, easy to obtain, and biodegradable, without secondary pollution. The prepared hydrogel delivers a cationic dye removal performance comparable to conventional starch adsorbents and exhibits acceptable cyclic reusability. Therefore, this material and its rapid preparation technology hold tentative potential for further development toward industrial printing and dyeing wastewater treatment after pilot-scale and real wastewater verification. This study demonstrates a rapid method for preparing starch-based adsorptive materials using the industry equivalent of the internal mixer.

## Figures and Tables

**Table 1:** Adsorption performance comparison of different biopolymer-based hydrogels for methylene blue.

Adsorbent	Preparation Method	Maximum Adsorption Capacity (mg g^−1^)	Reference
Xylan-gelatin cross-linked reusable hydrogel	Chemical cross-linking (30–40) min	26.04	[32]
Cellulose–attapulgite hydrogel	Chemical cross-linking (4 h)	24.3	[33]
Starch–humic acid hydrogel (This work)	Internal mixer (5 min)	9.6	Present study
Cross-linked porous starch	Chemical cross-linking (3 h)	9.46	[34]

**Table 2:** Adsorption kinetic parameters of CH20 for methylene blue.

Model		Parameters	Value
Pseudo-first Order Model	Nonlinear fitting	k_1_ (min^−1^)	0.05332
		R^2^	0.96224
		χ^2^	0.21408
Pseudo-second Order Model	Nonlinear fitting	k_2_ (g mg^−1^ min^−1^)	0.01475
		R^2^	0.9855
		χ^2^	0.08223
Liquid Film Diffusion (Initial stage)	linear fitting	k*_fd_* (h^−1^)	0.1665
		R^2^	0.9712
Intraparticle Diffusion (Stage 1)	linear fitting	k*_id_*_1_ (mg g^−1^ h^0.5^)	2.0504
		C_1_ (mg g^−1^)	−0.07819
		R^2^	0.9834
Intraparticle Diffusion (Stage 2)	linear fitting	k*_id_*_2_ (mg g^−1^ h^0.5^)	1.1802
		C_2_ (mg g^−1^)	2.795
		R^2^	0.9054

**Table 3:** Isotherm parameters for methylene blue adsorbed onto CH20.

Isotherm Model	Parameter	35 °C	40 °C	50 °C
Langmuir	Q_m_ (mg g^−1^)	19.53	19.96	20.53
	b (L mg^−1^)	0.071	0.411	1.265
	R^2^	0.982	0.985	0.987
Freundlich	K_F_	2.16	2.44	2.77
	1/n	0.42	0.39	0.36
	R^2^	0.995	0.996	0.997
D-R	Q_m_ (mg g^−1^)	9.49	14.60	16.82
	E (kJ mol^−1^)	0.67	0.66	0.64
	R^2^	0.981	0.983	0.985
Sips	Q_m_ (mg g^−1^)	19.41	19.82	20.37
	n_s_	1.38	1.46	1.56
	K_s_	0.068	0.076	0.085
	R^2^	0.992	0.994	0.995

## Data Availability

The original contributions presented in this study are included in the article. Further inquiries can be directed to the corresponding author.
